# Molecular ion formation on activated field emitters in atmospheric pressure field desorption mass spectrometry

**DOI:** 10.1007/s00216-023-04652-4

**Published:** 2023-03-24

**Authors:** Matthias Hoyer, Jürgen H. Gross

**Affiliations:** Institute of Organic Chemistry, Heidelberg University, Im Neuenheimer Feld 270, 69120 Heidelberg, Germany

**Keywords:** Field desorption (FD), Molecular ions, Atmospheric pressure ionization (API), Activated field emitter, Fourier transform-ion cyclotron resonance (FT-ICR), Ionization process

## Abstract

Atmospheric pressure field desorption (APFD) mass spectrometry (MS) has recently been explored as a new contribution to the field of ambient desorption/ionization (ADI). Depending on the selected polarity applied to the field emitter, ionic and polar analytes were demonstrated to deliver positive as well as negative ions. Whereas this recent study solely reported on the formation of even-electron ions of either polarity, the present work on APFD-MS demonstrates the abundant formation of positive molecular ions, M^+•^, from polycyclic aromatic compounds. Molecular ions were formed on and desorbed from standard 13-µm activated tungsten wire emitters at atmospheric pressure. The commercial field emitters were positioned at about 2 mm distance in front of the atmospheric pressure interface of a Fourier transform-ion cyclotron resonance (FT-ICR) mass spectrometer and the entrance electrode of the interface was set to –4.5 to –5.5 kV with respect to the emitter. Emitter-disrupting electric discharges did normally not occur under these conditions. The electric field strengths achieved at the dendritic microneedles were sufficient to allow for the abundant formation of M^+•^ ions of various polycyclic aromatic compounds such as benzo[a]pyrene, anthracene, fluoranthene, 1,1,4,4-tetraphenyl-butadiene, and 1-aza-[6]helicene. In case of the extremely basic 1-aza-[6]helicene protonation strongly competed with molecular ion formation and tended to suppress the field ionization process. All molecular ion compositions were assured by accurate mass-based formula assignments.

**Graphical Abstract:**

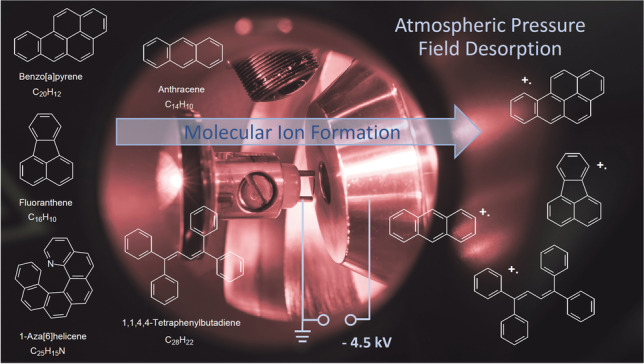

**Supplementary Information:**

The online version contains supplementary material available at 10.1007/s00216-023-04652-4.

## Introduction

Field ionization (FI) and field desorption (FD) are soft ionization techniques typically delivering intact positive molecular ions, M^+•^, or adduct ions like [M + H]^+^ and [M + alkali]^+^ of neutral molecular compounds [1–4]. The process of field ionization requires an electric field strength in the order of 1 V Å^–1^ to effect tunneling of an electron from the neutral analyte molecule toward the field emitter [1, 5, 6]. The established way to achieve the electric field strength for M^+•^ ion formation is to use activated field emitters, i.e., tungsten wires of 10–13 µm in diameter covered with field-enhancing microneedles [7–9], and to apply voltages in the order of 10 kV with respect to a counter electrode at about 2 mm distance.

Field desorption of preformed ions only requires field strengths in the order of 0.01 V Å^–1^ [5, 10, 11] rendering FD as well suited for the analysis of ionic compounds. In case of salts, [Cat^+^An^–^], FD delivers the intact cations, Cat^+^, frequently accompanied by cluster ions [Cat_n+1_An_n_]^+^ while polar molecules yield [M + H]^+^ or [M + alkali]^+^ ions [10, 12, 13]. As long as preformed ions are available on the emitter surface, FD does not require FI as an ionization process for ion desorption to occur. The ionization of neutrals, however, proceeds via FI of the molecules either on the surface or in case of higher volatility, probably in the gas phase, while still in close proximity to the emitter needle tips.

The modern variant of FD and FI, liquid-injection field desorption/ionization (LIFDI) [14–17], allows for sample introduction to the emitter under the complete exclusion of moisture and air [16–22].

All of these techniques, i.e., FI, FD, and LIFDI, are traditionally performed in high vacuum to suppress electric discharges due to the strong electric fields, because the extremely fragile activated tungsten wire emitters [8, 9] would otherwise be disrupted.

One route to allow for emitter potentials in the order of 10 kV while still avoiding electric discharges to the counter electrode is to perform FD at superatmospheric pressure (6 bars) [5]. Thus, bare 20-µm tungsten wire emitters set to 9–12 kV relative to a counter electrode at 1.6 or 5.0 mm distance, respectively, were used to generate positive ions of various ionic and highly polar compounds. This particular setup even permitted emitter heating and delivered FD spectra of high quality in terms of signal-to-noise ratio [5].

The very first use of activated tungsten field emitters at atmospheric pressure was documented as a side note in an article describing natural microscale emitters, i.e., among others the hairy legs of tiny insects [23]. In that work, standard activated emitters were employed as a control experiment and found to yield [M + H]^+^ ions of hexakis-(fluoroalkoxy)-phosphazenes from a commercial mass calibration mixture [24–26].

The relationship of field desorption of ionic compounds and microscale variants of electrospray ionization, e.g., nanoESI and probe electrospray ionization (PESI), was pointed out in a book chapter on PESI [27].

Increased reaction rates were observed in high electric fields generated by an FD emitter [6]. This work employed standard 13-µm activated tungsten wire emitters that were operated at the open atmosphere. When the emitters were set to 4–5 kV with respect to the counter electrode at 3–15 mm distance to the orifice of the API interface, positive ions, typically protonated molecules, [M + H]^+^, were formed. Nonetheless, in one instance, molecular ions, M^+•^, were also observed [6]. In this case, phenylhydrazone molecular ions, [C_6_H_8_N_2_]^+•^, were exclusively detected as long the emitter was positioned at 3 mm distance to the orifice of the API interface but disappeared in favor of [C_6_H_8_N_2_ + H]^+^ ions as soon as the emitter was retracted. The authors assigned this change to the decrease in electric field strength as field desorption of preformed ions occurs at field strengths two order of magnitude below those required for the field ionization process [5, 10–13, 28].

Inspired by the above findings, atmospheric pressure field desorption (APFD) using standard activated 13-µm tungsten wire emitters has recently been explored [29], more or less as a complementary approach to the vast field of ambient desorption/ionization (ADI) techniques [30–32]. In this study on APFD, the formation of positive and, for the first time, negative ions has been described [29]. Until then, reports on negative-ion FD had not only been scarce but also been restricted to high vacuum conditions [22, 33, 34]. Moreover, all ions described in this preceding work on APFD-MS were even-electron species, i.e., either the respective cations or anions of ionic liquids along with their corresponding cluster ions or protonated and deprotonated species formed from highly polar analytes [29]. In this work, a continuous transition from the FD process to the microscale ESI process had been discussed as in the aforementioned chapter on PESI [27].

As hitherto, molecular ion formation in APFD has only been reported in a singular case [6], this work explores the formation of molecular ions, M^+•^, of aromatic compounds from activated tungsten field emitters at atmospheric pressure and under ambient conditions.

## Materials and methods

### Mass spectrometer

APFD experiments were performed using a Bruker Apex-Qe Fourier transform ion cyclotron resonance (FT-ICR) mass spectrometer (Bruker Daltonics, Bremen, Germany) equipped with a 9.4 T superconducting magnet and an ESI-to-MALDI switchable Dual Source MTP. This mass spectrometer allows for tandem MS by mass-selection of precursor ions in a linear quadrupole (Q) situated in front of the FT-ICR analyzer. For tandem MS, precursor ions were selected in Q and activated by collision-induced dissociation (CID) with the argon buffer gas in an RF-only hexapole collision cell (h2) by applying an offset voltage.

The instrument was controlled by the Bruker ApexControl software (V 3.0.0), and data analysis was performed using the Bruker DataAnalysis software (V 4.3).

Before ICR mass analysis ions were accumulated for 0.5–2.0 s in the RF-only hexapole (h2). Ions were excited and detected using standard settings from previous work [29, 35, 36]. External mass calibration was established in positive-ion ESI mode using Agilent Tune Mix (G1969-85,000) [24–26] and generally delivered a mass accuracy in the order of 1–2 ppm.

APFD-FT-ICR mass spectra shown here were obtained by accumulation of 16 transients of either 1 M or 512 k data points. When the range *m/z* 130–1500 was selected, a 512 k data points transient resulted in a resolving power of *R* = 63,000 at *m/z* 358, and 1 M data points transient resulted in a resolving power of *R* = 125,000 at *m/z* 358, respectively.

### Atmospheric pressure FD ion source and operation

The APFD setup used and its general operation have recently been described in detail [29]. In brief, the emitter holder of a Bruker nanoESI source, mounted onto an *x*,*y*,*z*-adjustable sample stage, was replaced by a custom-built aluminum piece to clamp the FD emitter. The built-in CCD camera and a monitor were used to observe the emitter positioning process. The nanoESI source was grounded, while high voltage was exclusively applied to the counter electrode provided by the API interface. High voltage was adjusted via the settings provided by the API source controls. Settings at the upper technical limit of this source of –6 kV would result in spark discharges at the chosen distance of the emitter to the counter electrode, i.e., in this setup to the pair comprising spray shield and capillary cap. Thus, the voltages where normally set to –4.0 to –4.8 kV at the spray shield and to –4.5 to –5.5 kV at the cap underneath the spray shield. When, in rare circumstances, spark discharges were observed via the observation optics, the voltages were either slightly lowered or the emitter was retracted by about 0.5 mm.

**Fig. 1:**
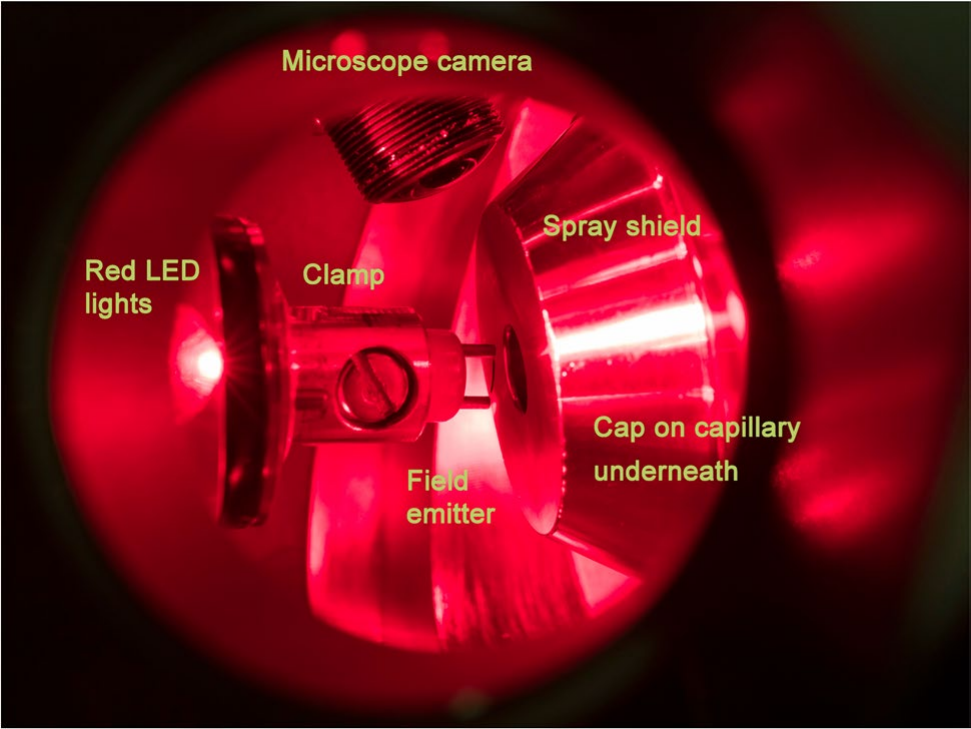
Configuration to align the activated emitter in front of the counter electrode provided by the so-called spray shield of the Bruker API interface. The photograph was taken through the right CCD camera port after unmounting this camera. At the top in the background, the left side CCD microscope camera is visible. The emitter-holding clamp is on the left side and positions the emitter in front of the center hole of the spray shield of the API interface. High voltage is only applied to the spray shield and to the entrance cap hidden underneath, whereas the emitter side is always kept at ground potential

All experiments described here used what was described as C2 in [29], i.e., the conventional ESI interface with the so-called spray shield and a rounded metal cap (orifice 0.90 mm in diameter) mounted on the glass transfer capillary (orifice 0.50 mm in diameter) underneath (Fig. 1).

The activated field emitters were of the standard type commercially available for the JEOL AccuTOF series of instruments [33, 37] and were based on 13-µm tungsten wires (Linden CMS, Weyhe, Germany).

The nebulizer gas for ESI was switched off at all times, the drying gas was either off or set to 1.2–2.0 l min^–1^ at 80–120 °C. Other instrument settings were the same as in ESI operation.

The samples were manually delivered to the emitter as solutions at 0.5–2.0 mg ml^–1^ by using a 10-µl syringe while the emitter was clamped into the emitter holder. The emitter was then mounted to the nanoESI source. After the runs, the emitter was rinsed with solvent to remove excessive analyte. An emitter could be used for several tens of acquisitions.

Some additional control experiments were also performed by direct analysis in real time (DART) using the same FT-ICR mass spectrometer in DART mode as previously described [38, 39] and by LIFDI using the JEOL AccuTOF instrument with a dedicated LIFDI source as communicated earlier [37].

### Chemicals

**Fig. 2:**
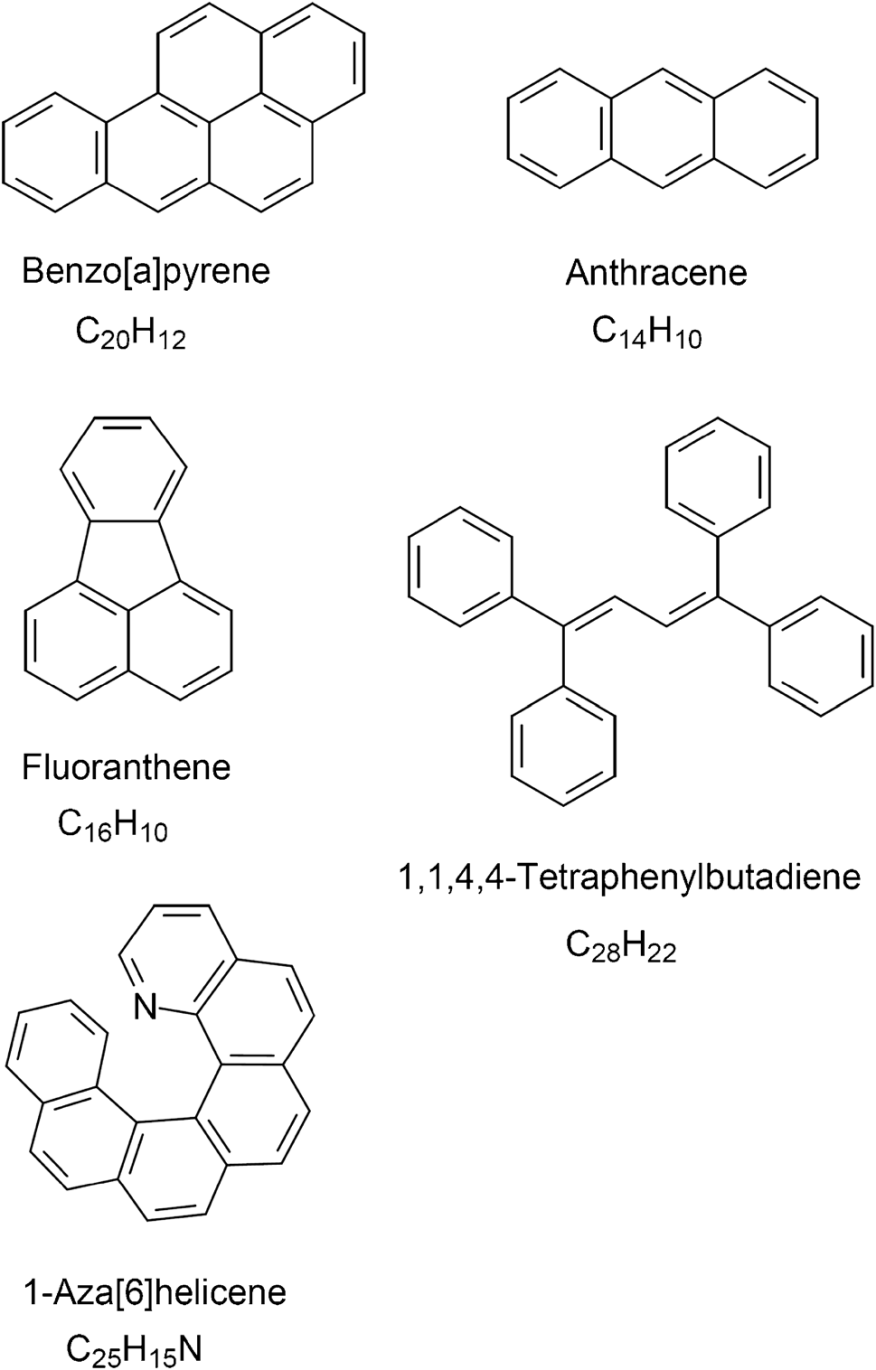
Compilation of analytes used in this study

The compounds used are listed in Table 1 along with their ionization energies and proton affinities [40, 41] and structures are shown in Fig. 2. Anthracene (CAS 120–12-7) and benzo[a]pyrene (CAS 50–32-8) were from Sigma-Aldrich (Steinheim, Germany), 1,1,4,4-tetraphenylbutadiene (CAS 1450–63-1) from Fluka (Buchs, Switzerland), fluoranthene (CAS 206–44-0) from Bruker Daltonics (Bremen, Germany), and 1-aza[6]helicene was preserved from a previous study [42]. The sample had originally been obtained as a gift for mass spectral studies [43–45]. Solvents were from Sigma-Aldrich or Fluka.

**Table 1:** Compounds analyzed by APFD-MS in the order of appearance. Ion energetic values were taken from Refs. [40, 41]

Compound name	Molecular ion formula and accurate *m/z*	Ionization energy [eV]	Proton affinity [kJ mol^–1^]
Benzo[a]pyrene	[C_20_H_12_]^+•^, 252.0934	7.12	890.0
Fluoranthene	[C_16_H_10_]^+•^, 202.0777	7.9	828.6
Anthracene	[C_14_H_10_]^+•^, 178.0777	7.44	877.3
1,1,4,4-Tetraphenylbutadiene	[C_28_H_22_]^+•^, 358.1716	-	-
1-Aza-[6]helicene	[C_25_H_15_N]^+•^, 329.1199	-	1000

## Results and discussion

### Benzo[a]pyrene

Based on its low ionization energy of 7.12 eV (Table 1) benzo[a]pyrene was selected as the first candidate to explore molecular ion formation in APFD. Whether benzo[a]pyrene would form molecular ions under ambient conditions and whether these would be stable enough to be transferred into the FT-ICR cell of the mass analyzer was probed in positive-ion DART mode. A DART helium temperature of 150 °C was found to deliver DART spectra of good intensity showing peaks of both the molecular ion, [C_20_H_12_]^+•^, *m/z* 252.0926 (calc. *m/z* 252.0934), and the [C_20_H_12_ + H]^+^, ion at *m/z* 253.1004 (calc. *m/z* 253.1012). Under standard DART-FT-ICR conditions with argon buffer gas in the accumulation hexapole (h2) in front of the ICR cell [38, 39], the intensity of the M^+•^ ion signal relative to the [M + H]^+^ ions was in the 25% range. Based on experience with softer thermalization in h2 by using helium buffer gas instead, the DART experiments were also performed while admitting helium in h2 [36]. However, there was only a mild effect causing the M^+•^ ion peak of benzo[a]pyrene relative intensity to rise to about 30% (see Electronic Supplementary Material, Fig. S1). For comparison, in conventional vacuum LIFDI, benzo[a]pyrene was found to deliver exclusively M^+•^ ions accompanied by [2 M]^+•^ cluster ions (Fig. S2).

**Fig. 3:**
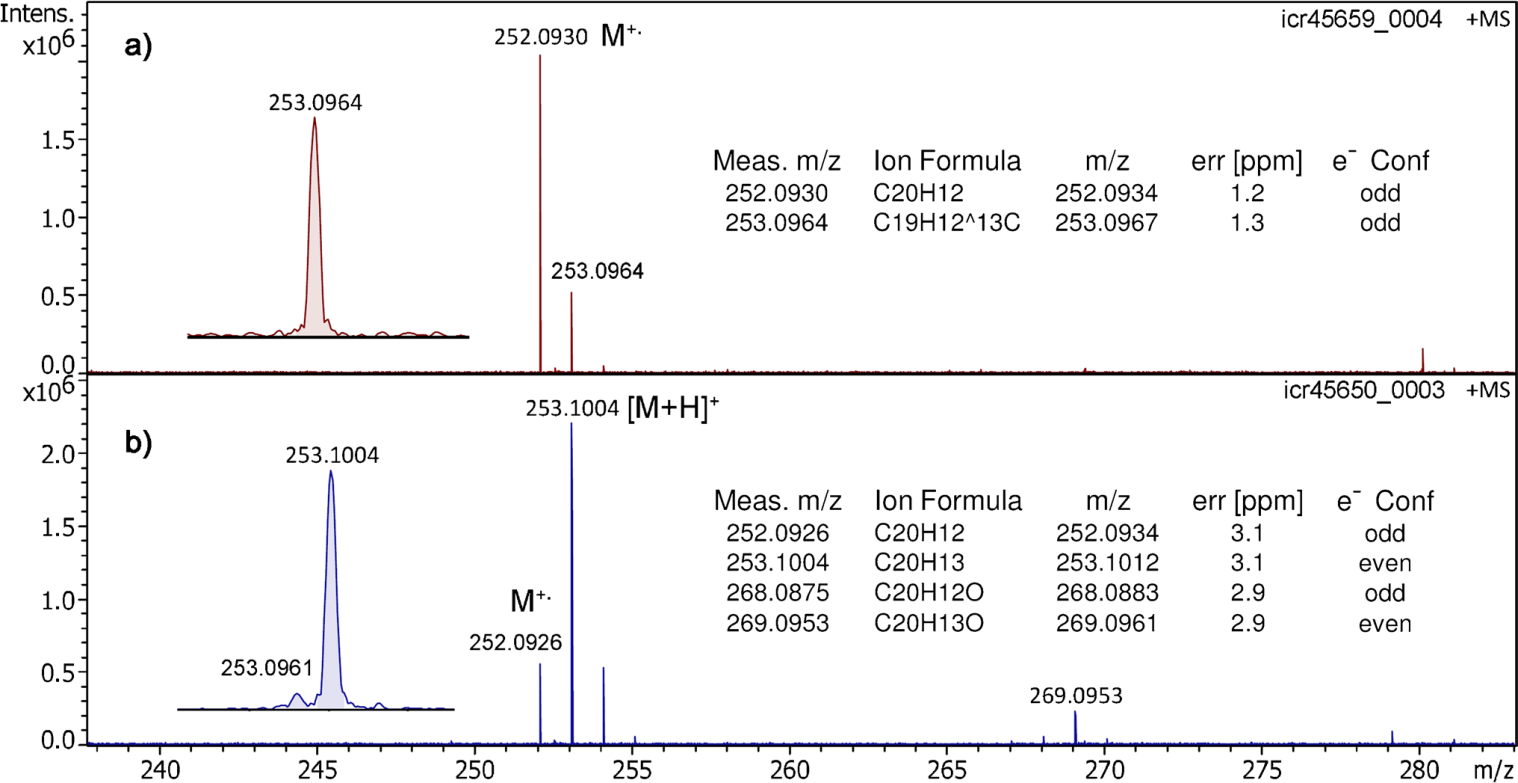
In **a** the positive-ion APFD spectrum of benzo[a]pyrene shows the molecular ion signal, *m/z* 252.0930, while in **b** the DART spectrum exhibits the M^+•^ ion peak at *m/z* 252.0926 accompanied by a four times more intensive [M + H]^+^ ion peak at *m/z* 253.1004. The insets provide expanded views of the peaks at *m/z* 253 demonstrating the separation of ^13^C isotopologs of the M^+•^ ions and protonated molecules (*left*) and list the formulas assigned to the ions (*right*)

The APFD spectrum of benzo[a]pyrene exhibited the molecular ion, [C_20_H_12_]^+•^, *m/z* 252.0930, as the only signal (Fig. 3). With 1 µl of benzo[a]pyrene at 1 mg ml^–1^ in acetone applied to the emitter, the spray shield set to –4.5 kV and the capillary entrance to –5.0 kV, a long-lasting M^+•^ ion signal having an intensity in the order of 1.5 × 10^6^ counts was produced. Thus, a single application of sample delivered a series of five replicate APFD spectra corresponding to a total ion production period of about 3 min (Fig. S3). In contrast to LIFDI, the [2 M]^+•^ cluster ion peaks were not observed in APFD. Nonetheless, protonation did not play a role in APFD as can be seen from the isotopic pattern of the molecular ion signal revealing pure M^+•^ ion formation (Fig. 3). In case of [M + H]^+^ ion formation, the ^13^C isotopic peak at *m/z* 253.0964 (calc. for [^13^CC_19_H_12_]^+•^ *m/z* 252.0967) would have been accompanied by a signal at *m/z* 253.1012 (calc. for [C_20_H_13_]^+^), a difference that has been well resolved at *R* = 175,000.

Thus, APFD did not only form molecular ions of benzo[a]pyrene, but like LIFDI, it also delivered them as the only ionic species. These findings provide evidence for the fact that the field ionization process can occur under ambient conditions, i.e., the field strength achieved here can at least result in FI of molecules of low ionization energy. Furthermore, as the signal intensity in APFD and DART was found to be about equal while the signal generated from 1 µg of sample in APFD lasted at least for 180 s as compared to about 15 s of desorption time obtained from 4 µg of sample in DART, the overall efficiency of APFD comprising ionization and transmission through the API interface can be estimated to be 45–50 times better than that of DART in this particular case.

At a later stage of this study an instrument tuning further optimized for ions of low *m/z* value even allowed benzo[a]pyrene molecular ion peak intensities of 1.4 × 10^8^ counts to be achieved. These spectra also revealed additional molecular ion signals at *m/z* 266.1089, [C_21_H_14_]^+•^, and at *m/z* 280.1246, [C_22_H_16_]^+•^, most probably due to minor impurities of the sample (Fig. S4). The occurrence of [C_21_H_14_]^+•^ and [C_22_H_16_]^+•^ ions indicated that M^+•^ ion formation was not restricted to benzo[a]pyrene as a singular case.

Another signal at *m/z* 250.0777, [C_20_H_10_]^+•^, was potentially caused by H_2_ loss from [C_20_H_12_]^+•^ rather than by a fourth compound. The high precursor ion intensity also allowed for essentially monoisotopic selection of the [C_20_H_12_]^+•^ ion that did not undergo notable fragmentation below a collision offset voltage of 30 V. Then, as expected for a PAH-type compound, losses of H_2_ and C_2_H_2_ occurred. These fragments at *m/z* 250.0775 and *m/z* 226.0776 corresponded to further radical ion species, i.e., to [C_20_H_10_]^+•^ by loss of H_2_ and [C_18_H_10_]^+•^ by loss of C_2_H_2_, respectively (Fig. S4).

### Fluoranthene and anthracene

Having an ionization energy of 7.9 eV, fluoranthene, C_16_H_10_, 202 u, was assumed to present another candidate for molecular ion formation in APFD. As the ApexQe mass spectrometer used here showed a quite sharp low-mass cut-off in the *m/z* 170–180 range, the transmission expected for the fluoranthene M^+•^ ion would already be reduced by some degree.

In vacuum LIFDI, fluoranthene delivered rather low M^+•^ ion intensity (Fig. S5). As indicated by the maximum of *m/z* 202 signal intensity immediately at the beginning of the acquisition, this was attributed to increased volatility, and thus, sublimation of the analyte before the emitter high voltage was fully turned on and the acquisition started.

**Fig. 4:**
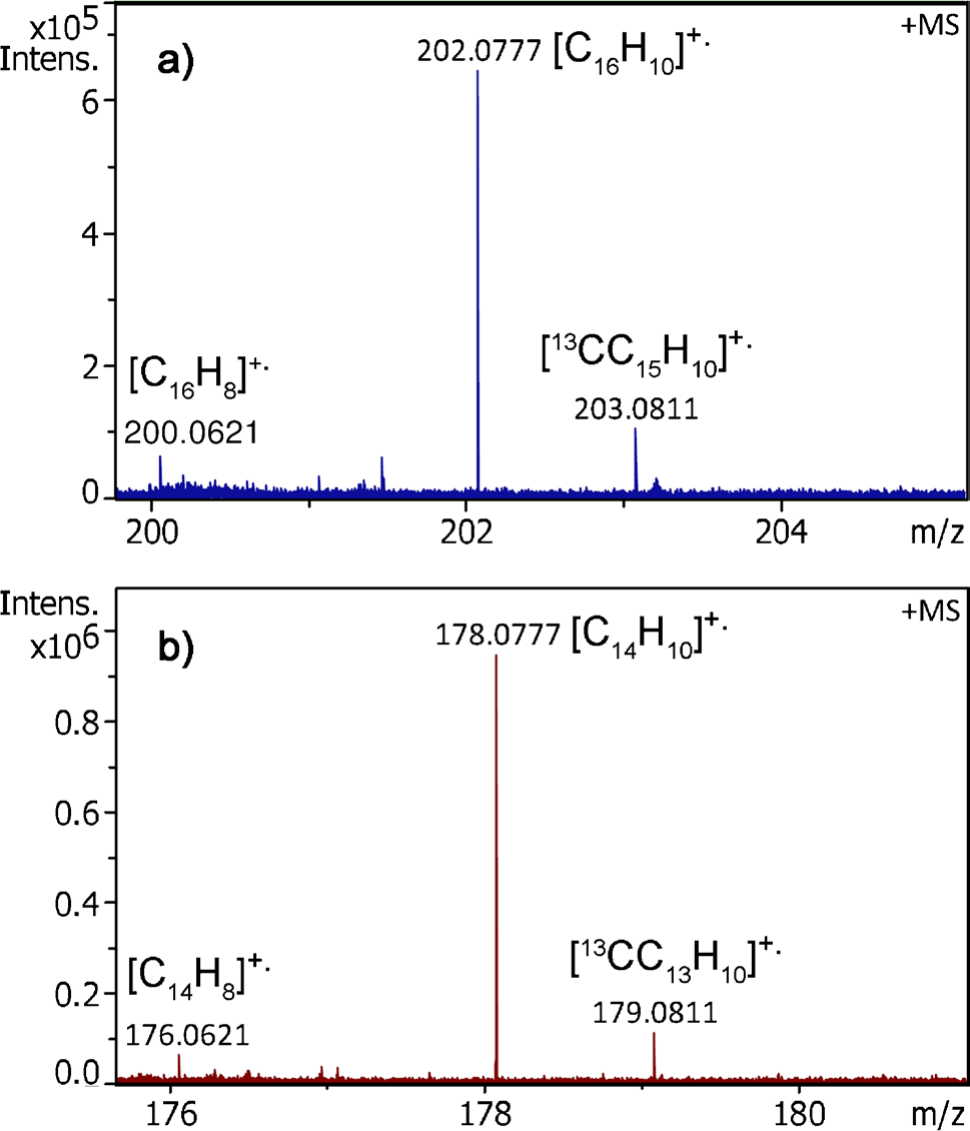
Molecular ion regions of positive-ion APFD spectra of **a** fluoranthene and **b** anthracene. In **a** the signals corresponding to the M^+•^ ion, [C_16_H_10_]^+•^, *m/z* 202.0777, the first isotopic ion [^13^CC_15_H_10_]^+•^, *m/z* 203.0811, and a [C_16_H_8_]^+•^ fragment, *m/z* 200.0621, by loss of H_2_ are observed. In **b** the signals due to the M^+•^ ion, [C_14_H_10_]^+•^, *m/z* 178.0777, the first isotopic ion [^13^CC_13_H_10_]^+•^, *m/z* 179.0811, and a [C_14_H_8_]^+•^ fragment, *m/z* 176.0621, by loss of H_2_ do occur. The M^+•^ ion in spectrum **b** required the presence of residual benzo[a]pyrene on the emitter to appear

In APFD the fluoranthene M^+•^ ion signal could only be obtained under optimal conditions, i.e., with either a fresh emitter and/or close to the upper limit of the ion source potentials (Fig. S6). It turned out that the mediocre intensity (ca. 2 × 10^5^ counts) was in part due to higher volatility of the sample. Thus, sample loss could substantially be reduced when the desolvation gas temperature was lowered to 80 °C. Under these conditions, the signals of the fluoranthene M^+•^ ion, [C_16_H_10_]^+•^, *m/z* 202.0776 (*m/z* 202.0777 calc. for [C_16_H_10_]^+•^), and the first isotopic ion [^13^CC_15_H_10_]^+•^, *m/z* 203.0811, were reproducibly observed at about 6 × 10^5^ counts, thereby yielding the next example of molecular ion formation in APFD (Fig. 4).

Anthracene, C_14_H_10_, 178 u, with its lower ionization energy of just 7.44 eV was expected to serve as an even more suited candidate for molecular ion formation in APFD. As far as signal intensity and volatility were concerned, in LIFDI, anthracene behaved analogous to fluoranthene (Fig. S7). Unfortunately, the anthracene M^+•^ ion peak at *m/z* 178.0777 (calc. for [C_14_H_10_]^+•^) would appear directly at the lower *m/z* limit of the ApexQe mass spectrometer. A rather weak signal of the anthracene molecular ion was in fact observed at *m/z* 178.0777 (*m/z* 178.0777 calc. for [C_14_H_10_]^+•^) accompanied by the first isotopic ion [^13^CC_13_H_10_]^+•^, *m/z* 179.0811. While both ions were clearly discernible, even cross contamination from benzo[a]pyrene from previous runs still tenfold exceeded their intensity (Fig. 4).

Trials to get anthracene M^+•^ ions in the absence of benzo[a]pyrene failed. It was therefore assumed that benzo[a]pyrene had exerted a positive effect, presumably by slowing down the sublimation of anthracene. In fact, the presence of the much less volatile benzo[a]pyrene moderated the loss of sample (Fig. S8).

APFD spectra of a mixture containing anthracene, fluoranthene, and benzo[a]pyrene at roughly equal concentrations were acquired to explore this aspect. While M^+•^ ion peaks of anthracene and fluoranthene showed up when sample was freshly applied, they almost disappeared in a second run started without adding new sample. This behavior was reproducible (Fig. S8). Overall, the spectra also indicated a by roughly two orders of magnitude lower sensitivity for anthracene and fluoranthene as compared to benzo[a]pyrene and a much quicker depletion of anthracene and fluoranthene while benzo[a]pyrene persisted. In fact, the molecular ion peak of benzo[a]pyrene was therefore established as an aid in emitter alignment and ion source tuning during the entire study.

### 1,1,4,4-Tetraphenylbutadiene

**Fig. 5:**
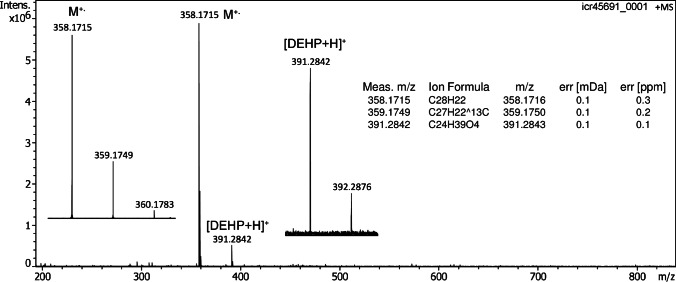
Positive-ion APFD spectrum of 1,1,4,4-tetraphenylbutadiene when 1 µl of sample at 1 mg ml^–1^ dichloromethane was applied to the emitter, the spray shield set to –4.8 kV and the capillary entrance to –5.3 kV. The molecular ion, [C_28_H_22_]^+•^, *m/z* 358.1715, was formed exclusively as shown by the expanded view of the molecular ion region (*left* insert). In addition, [DEHP + H]^+^ ions were observed at *m/z* 391.2842. The insert on the right provides the formula assignments

Having four phenyl groups connected via a conjugated -system, 1,1,4,4-tetraphenylbutadiene was also identified as a potential candidate for molecular ion formation in APFD. In positive-ion DART mode, this compound behaved similar to benzo[a]pyrene as it exhibited both M^+•^ and [M+H]^+^ ion peaks, however at about equal intensity [46]. As expected, with 1 µl of sample at 1 mg ml^–1^ in dichloromethane applied to the emitter, the spray shield set to –4.8 kV and the capillary entrance to –5.3 kV, the M^+•^ ion signal was detected at high intensity of 6 × 10^6^ counts (Fig. 5). Again, in APFD the M^+•^ ion peak showed up as the only signal related to the aromatic hydrocarbon. The peak at *m/z* 358.1715 (calc. for [C_28_H_22_]^+•^ *m/z* 358.1716) also proved to be long-lasting, allowing for several spectra in a row to be acquired from a single application of sample. In addition, the plasticizer di-(2-ethylhexyl)-phthalate (DEHP) contributed a [DEHP + H]^+^ ion signal at *m/z* 391.2842 (calc. for [C_24_H_39_O_4_]^+^ *m/z* 391.2843) to the spectrum. Obviously, this impurity with four oxygen atoms in its structure preferred protonation over radical ion formation. It was therefore assumed that compounds bearing heteroatoms as their basic sites would generally tend to yield even-electron ions while only compounds providing both quite low ionization energies and having no heteroatoms as basic sites should yield molecular ions of notable abundance via field ionization in APFD.

### 1-Aza[6]helicene

In contrast to the hydrocarbons examined so far, 1-aza[6]helicene is a polycyclic aromatic pyridine base [44, 45] with a known high proton affinity of 1000 kJ mol^–1^ [43]. This compound had been studied in laser desorption ionization (LDI) where laser desorbed neutrals were shown to undergo gas phase protonation with ions from electrospray within the ion source housing [42].

The LIFDI spectrum of 1-aza[6]helicene mostly showed M^+•^ ions, *m/z* 329.11. The occurrence of some protonated molecules, [M + H]^+^, at *m/z* 330.11 could only be revealed by comparison of the calculated ^13^C ion peak intensity (27.5%) and the experimental value of 34–36%, indicating 7–8% of [M + H]^+^ ion formation (Fig. S9).

**Fig. 6:**
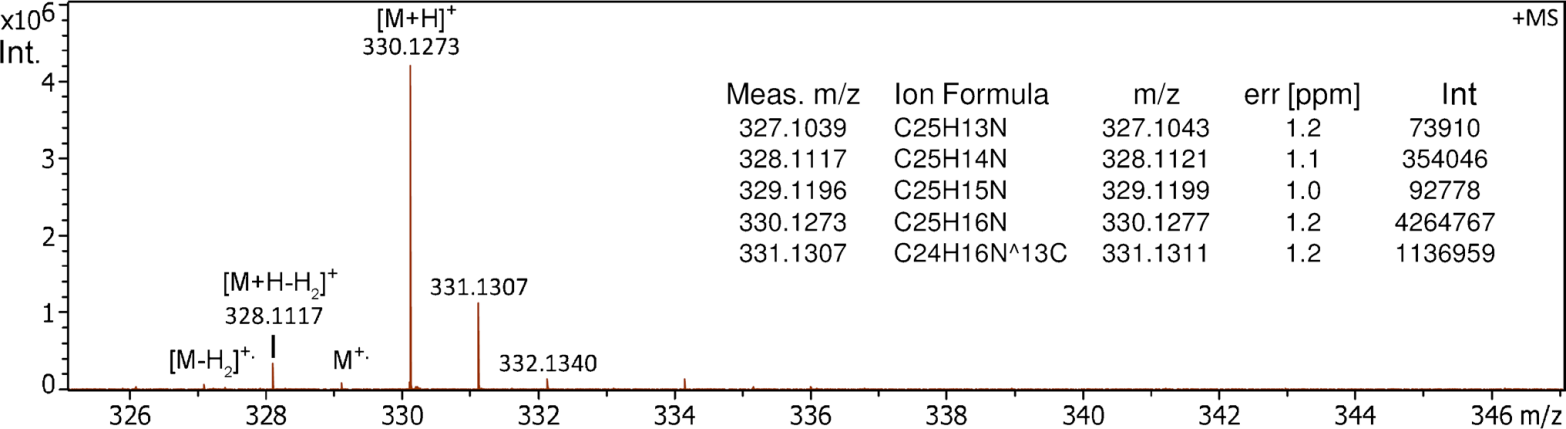
APFD spectrum of 1-aza[6]helicene acquired with –4.8 kV at the shield and –5.3 kV at the cap showing the [M + H]^+^ ion peak at *m/z* 330.1273 and the molecular ion signal at *m/z* 329.1196, here at a relative intensity of 2.3% of the [M + H]^+^ ion peak. The inset provides the formula assignments of all relevant species based on accurate mass data

In APFD, 1-aza[6]helicene showed an onset of [M + H]^+^ ion formation at *m/z* 330.1273 (*m/z* 330.1277 calc. for [C_25_H_16_N]^+^) at potentials as low as –3.6 kV at the shield and –4.1 kV at the cap. A first hint of the molecular ion at *m/z* 329.1196 (*m/z* 329.1199 calc. for [C_25_H_15_N]^+•^) only appeared at somewhat higher ion source potentials, i.e., at –3.9 kV at the shield and –4.4 kV at the cap (Fig. S10). Nonetheless, with the potentials set to the typical level of –4.8 kV at the shield and –5.3 kV at the cap, the [C_25_H_15_N]^+•^ ion relative intensity established at about 2.5% of the [M + H]^+^ ion (Fig. 6). Both types of ions underwent some loss of H_2_. However, while the [C_25_H_14_N]^+^ fragment ion peak only appeared at 8–9% intensity relative to the [M + H]^+^ ion peak, the [M–H_2_]^+•^ ion peak appeared at about 70% of the M^+•^ ion peak intensity, i.e., the radical ion clearly underwent fragmentation at a much higher rate, thereby further reducing its quite low relative intensity.

As already indicated by the [M + H]^+^ ion formation in case of DEHP, the formation of molecular ions suffered from very strong competition by protonation as soon as the analyte molecules offered heteroatoms as basic sites.

### Ionization mechanism

As far as the mechanism of ionization is concerned, the results indicate a true field ionization process, i.e., the withdrawal of an electron by action of very strong electric fields [1, 5, 6]. Concerning the strong increase of anthracene and fluoranthene molecular ions in the presence of benzo[a]pyrene (Fig. S8), one might speculate that molecular ions of the latter lead to those of the former via charge transfer. However, due to the higher ionization energies of anthracene (7.44 eV) and fluoranthene (7.9 eV), charge transfer from molecular ions of benzo[a]pyrene (7.12 eV) can be ruled out.

An APCI-like ionization pathway comes to mind as an alternative. In such a case, corona discharges at the tips of the emitter needles would cause a sequence of reactions as in APCI, DART, or other ADI techniques [30–32].

Consequently, the observed formation of both M^+•^ and [M + H]^+^ ions of benzo[a]pyrene and 1,1,4,4-tetraphenylbutadiene in DART would dictate that either species should also appear in APFD if an APCI-like mechanism was at work. However, pure molecular ion formation was observed in APFD of some polycyclic aromatic hydrocarbons. This strongly indicates a true FI process. The limited field strengths that can be applied here without electric discharges go along with a limitation in that only compounds of low ionization energy, roughly below 8 eV, would be ionized via the FI pathway. The field strength required for desorption of preformed ions from the condensed phase is far below that for FI [10–13, 28], and thus, the desorption of [M + H]^+^ ions can be expected to be dominant as soon as this pathway becomes feasible. This is in accordance with the behavior of DEHP and 1-aza[6]helicene reported above. Nonetheless, [M + H]^+^ ion formation is also known as a secondary process of molecular ions formed by FI [47, 48].

## Conclusion

This is the first systematic study of molecular ion formation in atmospheric pressure field desorption (APFD). APFD had recently been realized by adaptation of a nanoESI source to serve for precise emitter positioning in front of the atmospheric pressure interface of an FT-ICR mass spectrometer [29]. This setup enabled ion desorption from 13-µm activated tungsten emitters at atmospheric pressure and essentially under ambient conditions. Here, the application of the method has been expanded to polycyclic aromatic hydrocarbons. In APFD, these compounds formed abundant molecular ions via field ionization. The compositions of the ions were characterized by accurate mass, and in one case, the fragmentation pathways were examined by tandem MS.

The APFD setup employed here admittedly was quite simple yet effective and one might expect further improvements in ionization efficiency and ion transmission by the construction of more advanced emitter positioning and the implementation of emitter heating. It may also be doubted that APFD can effectively compete with top-of-the-line ADI techniques. Nonetheless, this work provided new insights in how ions may be formed under ambient conditions and in how FI and FD may be used in combination with instruments only providing atmospheric pressure ion sources.

## Supplementary Information

Below is the link to the electronic supplementary material.

Supplementary file1 (PDF 1841 KB)
