# Supplementary material for: Molecular ion formation on activated field emitters in atmospheric pressure field desorption mass spectrometry
Source: Anal Bioanal Chem. 2023 Mar 24;415(12):2307–15. doi: 10.1007/s00216-023-04652-4 (PMC10115680; doi:10.1007/s00216-023-04652-4)

Molecular ion formation on activated field emitters in atmospheric pressure field desorption-mass spectrometry

Supplementary Data

Matthias Hoyer, Jürgen H. Gross (ORCID 0000-0003-0748-2535)

Address

Institute of Organic Chemistry

Heidelberg University

Im Neuenheimer Feld 270

69120 Heidelberg

Germany

Correspondence to

email: juergen.gross@oci.uni-heidelberg.de

phone: +49/6221/54-8409

fax: +49/6221/54-4205

Fig. S1. Positive-ion DART-FT-ICR mass spectra of benzo[a]pyrene (1 mg ml^{-1} in acetone, 4 µl applied) at a DART helium temperature of 150 °C showing molecular ions, $[C_{20}H_{12}]^{+\bullet}$, and protonated molecules, $[C_{20}H_{12}+H]^{+}$, formed under ambient conditions. Switching the buffer gas in h2 from argon (0.6 l min^{-1}) to helium at two different flows (1.0 and 2.0 l min^{-1}) does not notably influence the intensity of the $M^{+\bullet}$ ions relative to the $[M+H]^{+}$ ions. They are in the 25–30 % range, i.e., helium might slightly improve the $M^{+\bullet}$ ion abundance. The formula assignments are provided along with the spectrum at the bottom. An oxidized species, probably a by-product of the DART process, is observed in addition (missing in LIFDI, cf. Fig. S2).

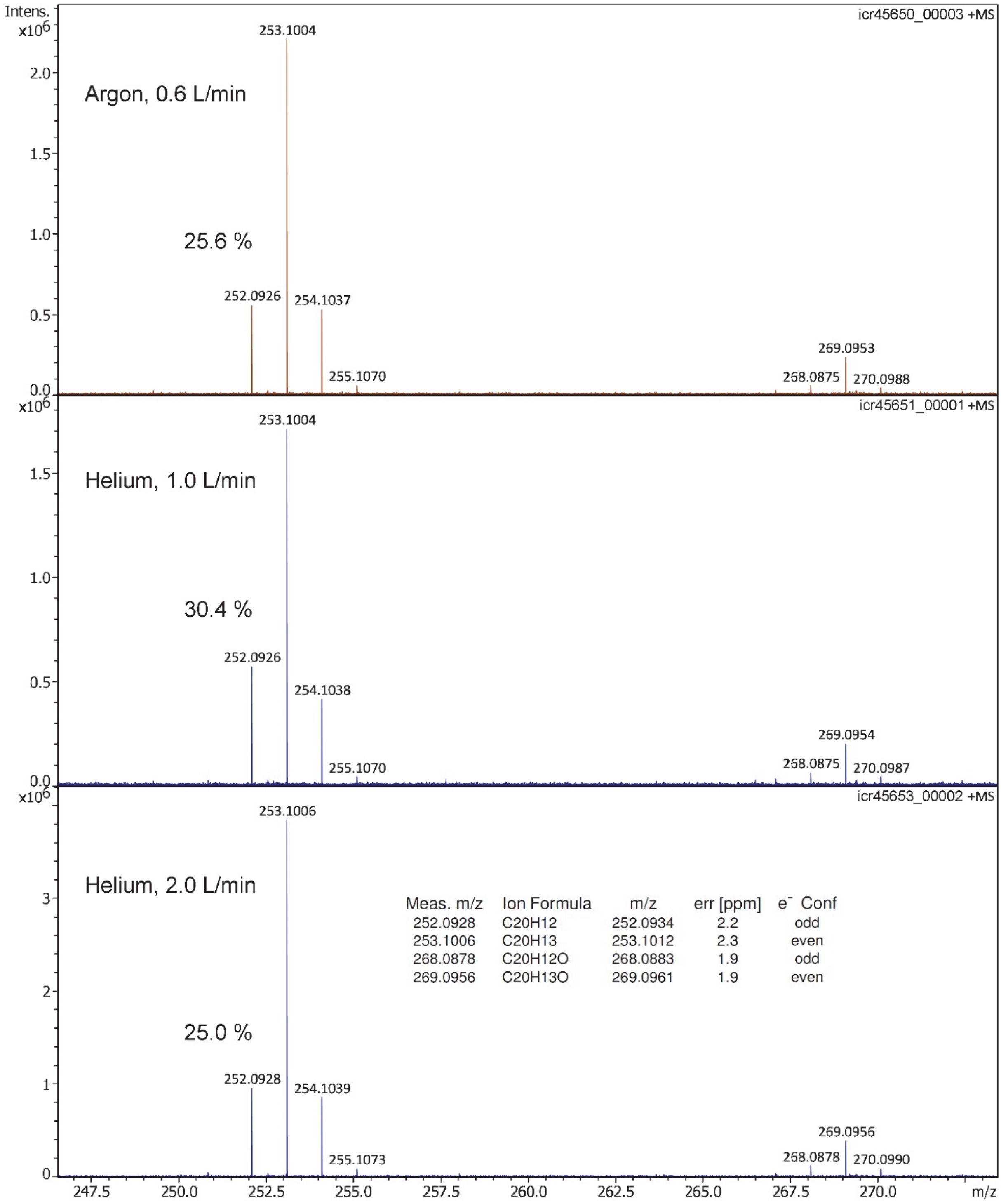

Fig. S2. In vacuum LIFDI, benzo[a]pyrene was found to deliver exclusively $M^{+\bullet}$ ions accompanied by $[2M]^{+\bullet}$ cluster ions. These spectra were obtained using the JEOL AccuTOF GCx mass spectrometer with the self-supplied LIFDI source*. A solution of benzo[a]pyrene at 1 mg ml^{-1} in acetone was transferred to the emitter, which was set to –10 kV and heated at 30 mA min^{-1}. The long-lasting signals could be observed from a few seconds after start **a)** 0.07–0.10 min, until about 1 min **b)** to finally **c)** acquired at 0.72–0.93 min. The selective formation of molecular ions is demonstrated by the expanded regions inserted in **c)**.

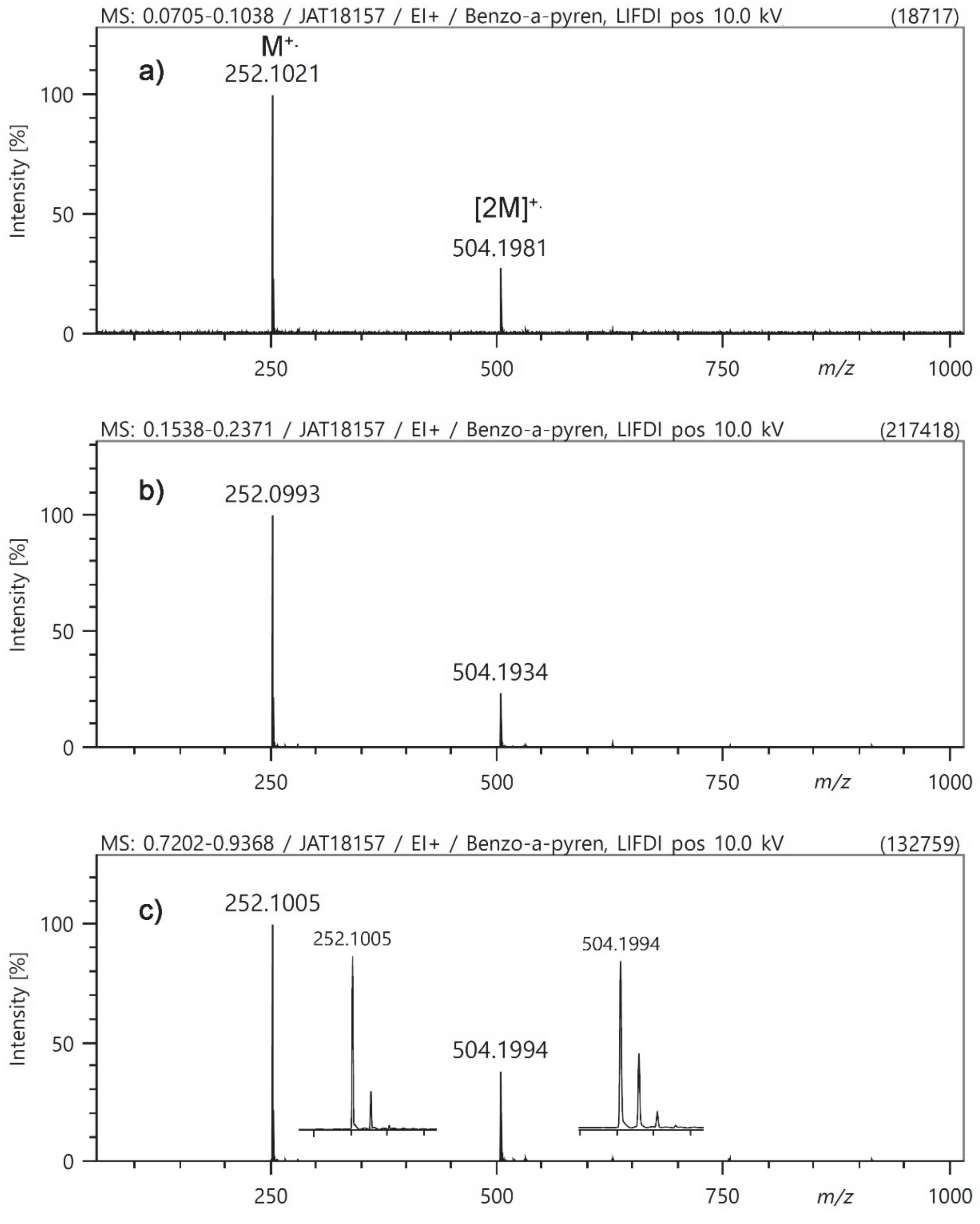

*M. H. Linden, H. B. Linden, N. Nieth, J. H. Gross, „Self-Supplied Liquid Injection Field Desorption/Ionization Ion Source for an Orthogonal Time-of-Flight Instrument", *J. Am. Soc. Mass Spectrom.* **2019,** 30, 2358-2368.

Fig. S3. Positive-ion atmospheric pressure field desorption (APFD) FT-ICR spectra of benzo-[a]pyrene (1 mg ml^{-1} in acetone, 1 µl applied, capillary –5.0 kV, shield –4.5 kV). All five replicates have been obtained in a series after a single application of sample onto the emitter. The fifths run has been started 170 s after the first, which corresponds to > 180 s of continuous ion desorption. The inserts display the expanded molecular ion regions revealing pure $M^{+\bullet}$ ion formation. In contrast to LIFDI, the $[2M]^{+\bullet}$ cluster ions are not observed. Formula assignments of the monoisotopic $M^{+\bullet}$ ion and first ^{13}C ion are provided along with the fourth spectrum. The other peaks of low intensity are mostly due to electronic noise.

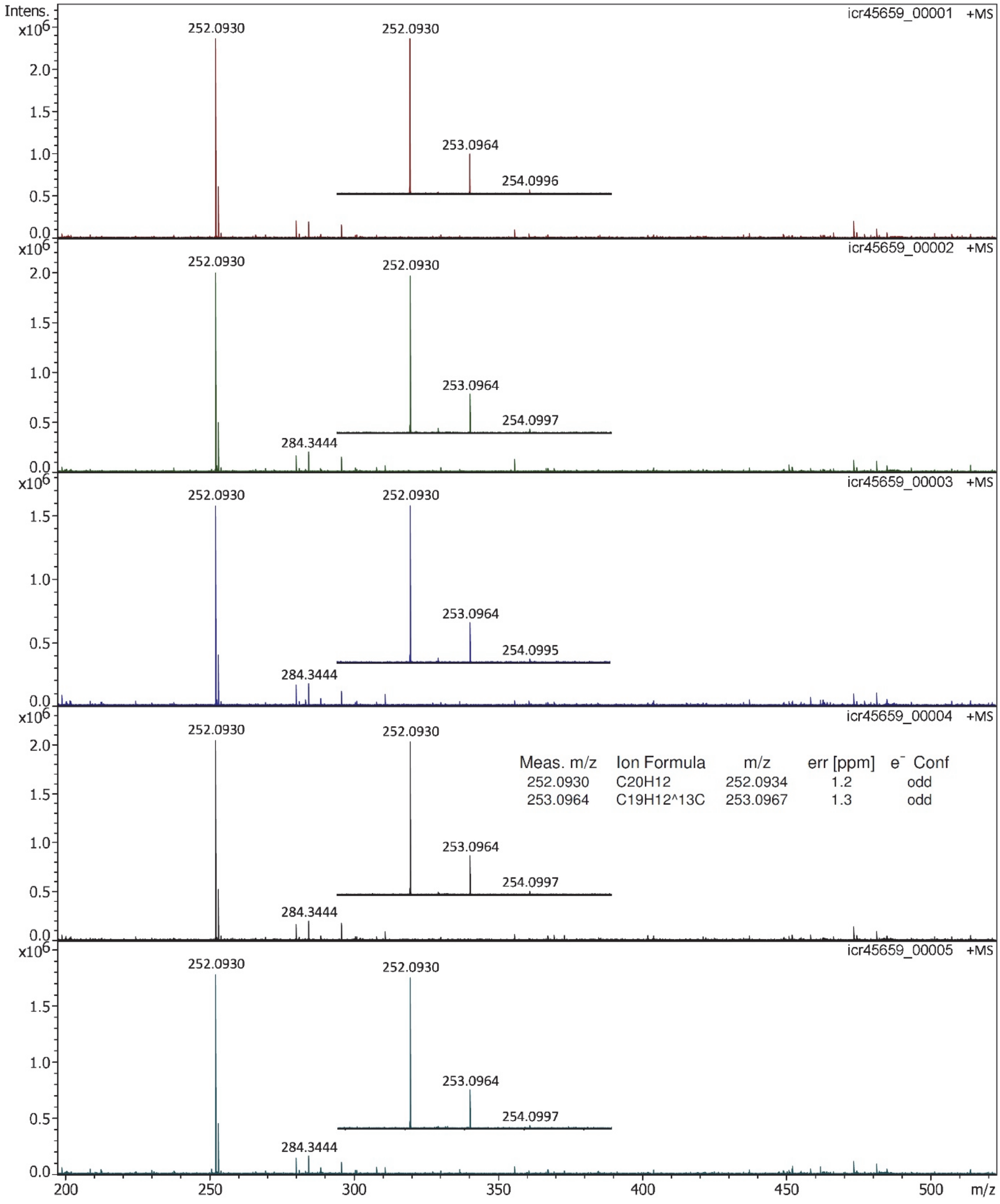

Fig. S4. Positive-ion APFD-FT-ICR spectra of benzo[a]pyrene (1 mg ml^{-1} in acetone, 1 µl applied, capillary –4.5 kV, shield –4.0 kV). All spectra have been obtained in a series after a single application of sample onto the emitter. The top spectrum shows the $M^{+\bullet}$ ion at an intensity of 1.4 x 10^{8} counts and reveals additional molecular ion signals at *m/z* 266.1089, $[C_{21}H_{14}]^{+\bullet}$, and at *m/z* 280.1246, $[C_{22}H_{16}]^{+\bullet}$, most probably due to minor impurities of the sample. It also permits monoisotopic precursor ion selection for tandem MS of $[C_{20}H_{12}]^{+\bullet}$. The $M^{+\bullet}$ ion starts to show notable fragmentation at a collision offset of 30 V. The fragments formed are additional radical ions, i.e., $[C_{20}H_{10}]^{+\bullet}$ by loss of H_2 and $[C_{18}H_{10}]^{+\bullet}$ by loss of C_2H_2.

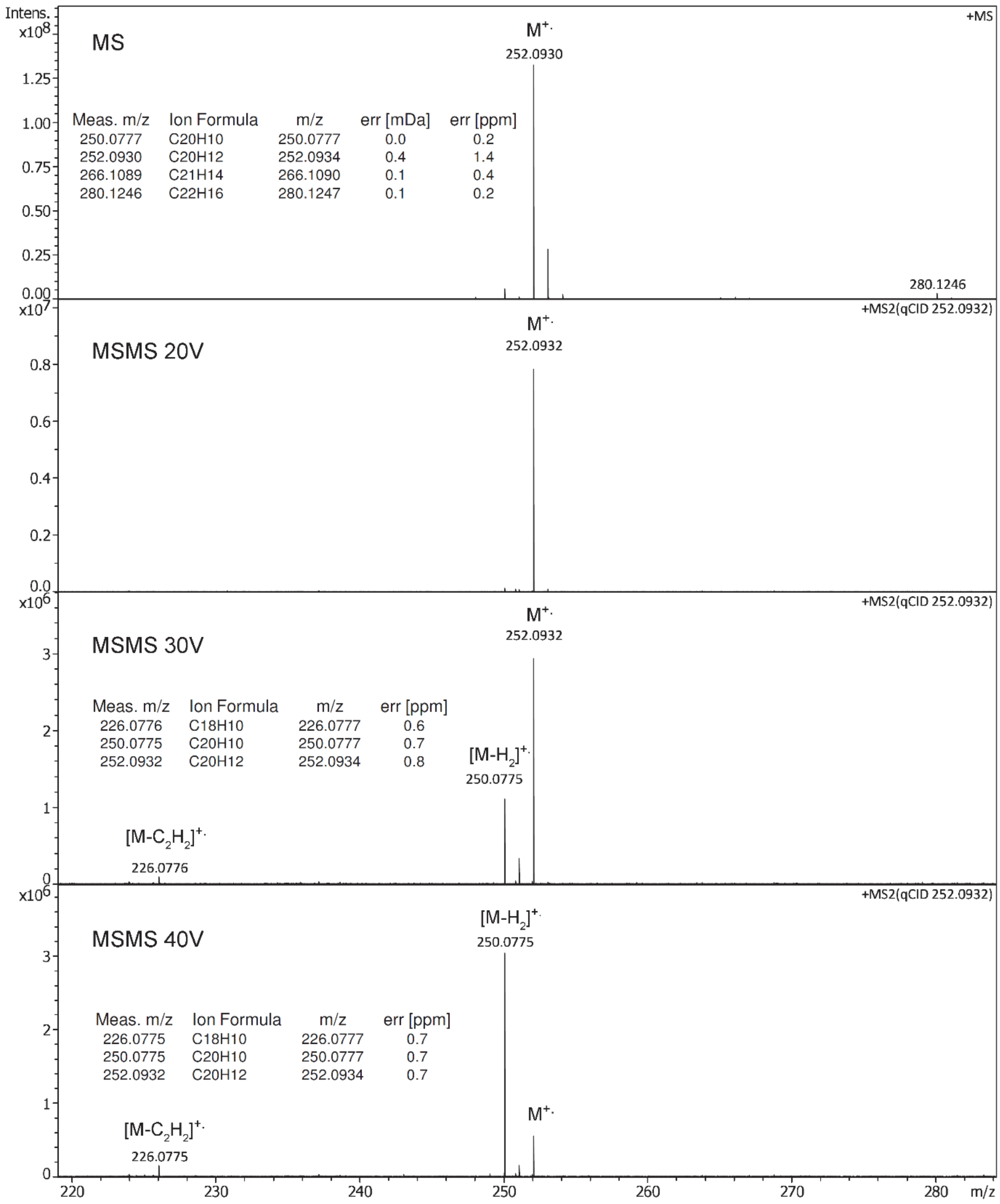

Fig. S5. In vacuum LIFDI, fluoranthene delivered exclusively $M^{+\bullet}$ ions, however at rather low intensity (*bottom*). As indicated by the maximum of *m/z* 202 early in the EIC (*top*), this could be attributed to sublimation of the analyte before the emitter high voltage was fully turned on and the acquisition started. LIFDI conditions were as in Fig. S2, i.e., a solution of fluoranthene at 1 mg ml^{-1} in toluene was transferred to the emitter, which was set to –10 kV and heated at 30 mA min^{-1}. The spectrum was acquired using the JEOL AccuTOF GCx.

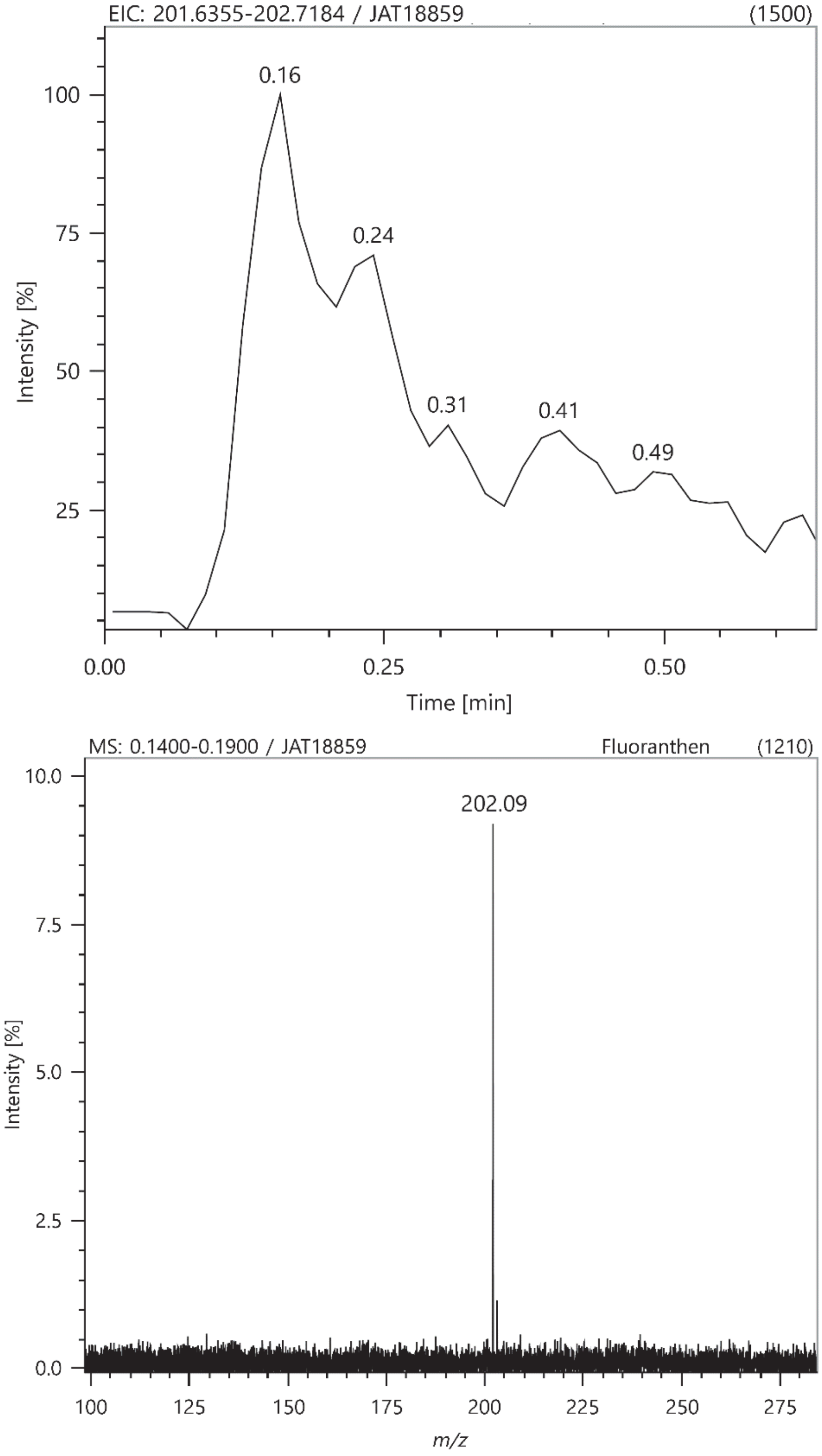

Fig. S6. Set of APFD spectra of fluoranthene **a)** with fresh emitter, shield at –4.5 kV and cap at –5.0 kV and **b)** on a different day with shield at –5.0 kV and cap at –5.5 kV, desolvation gas 120 °C at 1.5 l min^{-1}. The compositions of the monoisotopic molecular ion, *m/z* 202.0776, and of the first ^{13}C isotopic peak could be assured by their accurate mass. The intensity of the fluoranthene molecular ion is by orders of magnitude lower than that observed with benzo[a]pyrene. It turned out that the extremely low intensity was in part due to higher volatility of the sample.

Thus, sample loss was substantially reduced with the desolvation gas at 80 °C (cf. Fig. S8).

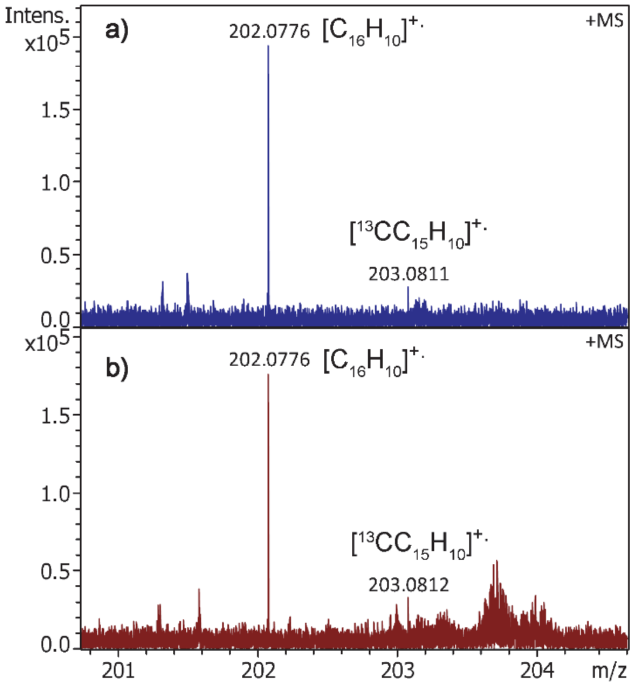

Fig. S7. In vacuum LIFDI, anthracene also delivered $M^{+•}$ ions that appeared at rather low intensity (*bottom*). As indicated by the sharp maximum of *m/z* 178 very early in the EIC (*top*), this could be attributed to sublimation of the analyte before the emitter high voltage was fully turned on and the acquisition started. LIFDI conditions were as in Fig. S2, i.e., a solution of anthracene at 1 mg ml^{-1} in toluene was transferred to the emitter, which was set to –10 kV and heated at 30 mA min^{-1}. The spectrum was acquired using the JEOL AccuTOF GCx.

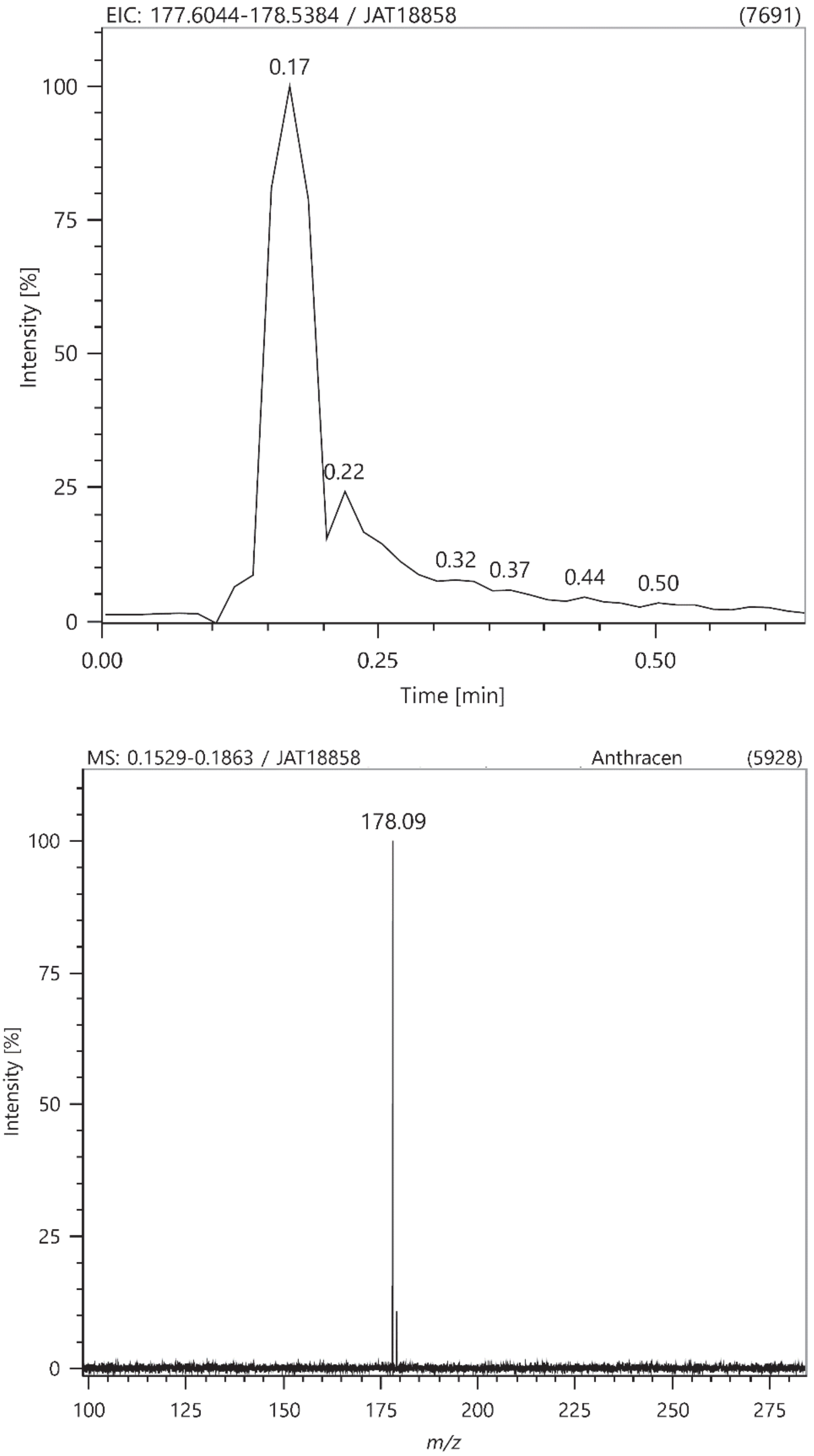

Fig. S8. APFD spectra of a mixture containing anthracene, fluoranthene, and benzo[a]pyrene at roughly equal concentrations. APFD settings were 1 µl solution applied, capillary –5.0 kV, shield –4.5 kV, desolvation gas 80 °C at 1.5 l min^{-1}. In **a)**, the first acquisition, sample was freshly applied, while in **b)**, the second run was started without adding new sample. The procedure was repeated in **c)** and **d)**. Inserts show the anthracene and fluoranthene region at expanded intensity scale and also provide formula assignments for anthracene and fluoranthene ions in **a)** and **c)**. The spectra indicate i) a by roughly two order of magnitude lower sensitivity for anthracene and fluoranthene as compared to benzo[a]pyrene and ii) a much quicker depletion of anthracene and fluoranthene while benzo[a]pyrene persists.

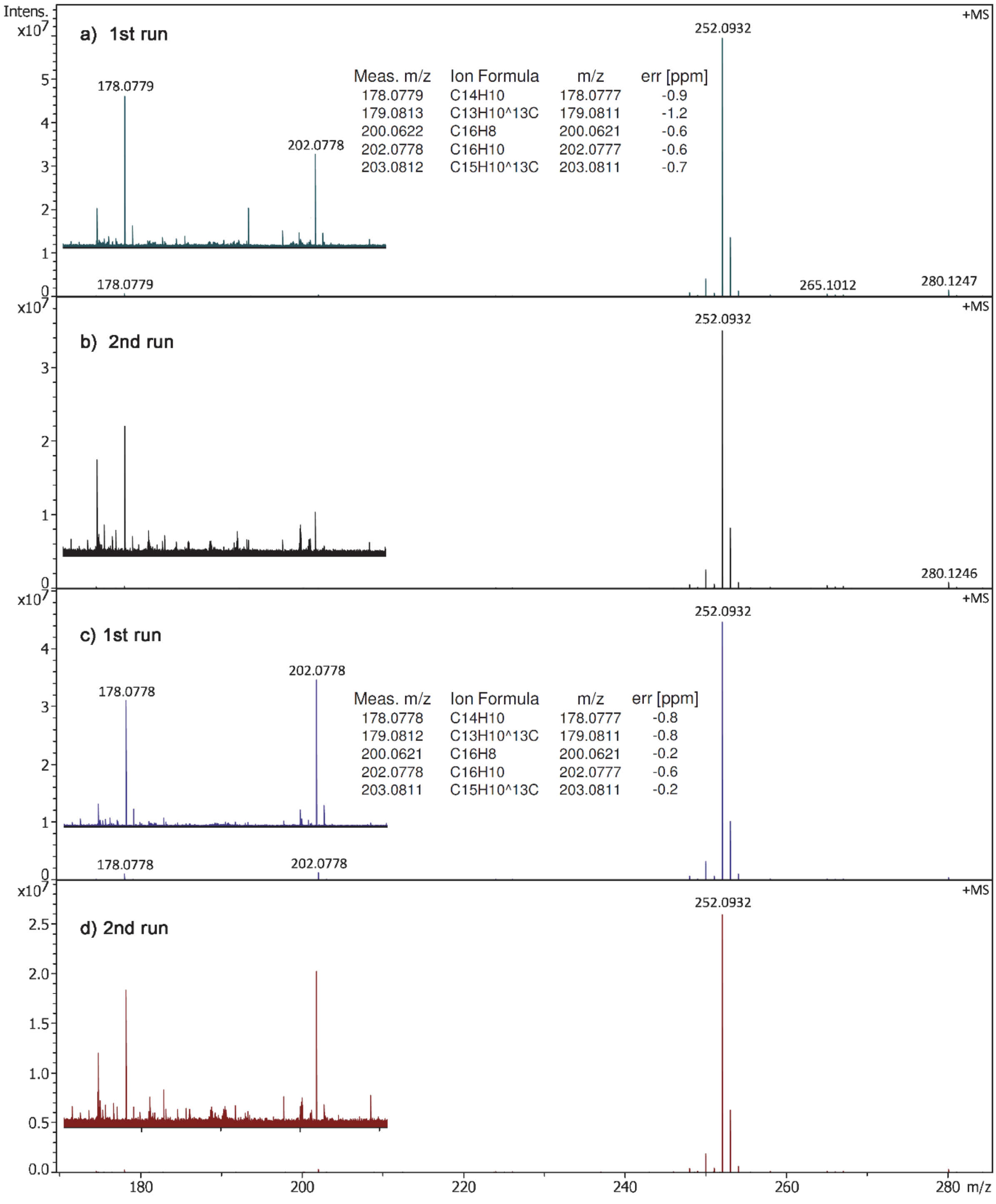

Fig. S9. LIFDI spectra of 1-aza[6]helicene. LIFDI conditions were as in Fig. S2, i.e., a solution of the sample at ca. 1 mg ml^{-1} in tetrahydrofuran was transferred to the emitter, which was set to –10 kV and heated at 30 mA min^{-1} and the spectrum was acquired using the JEOL AccuTOF GCx. In LIFDI, 1-aza[6]helicene mostly delivered $M^{+\bullet}$ ions, *m/z* 329.11, that appeared during an elongated period as visible from the EIC of *m/z* 329 (*top*). Spectra extracted from early and late sections during this period are shown (*middle* and *bottom*). Inserts show the expanded molecular ion regions of the LIFDI spectra. The compared to a calculated value of 27.5 % too high [M+1] ion intensities of 35.7 % and 34.2 %, respectively, indicate that about 7–8 % of the ions are formed as $[M+H]^+$ species.

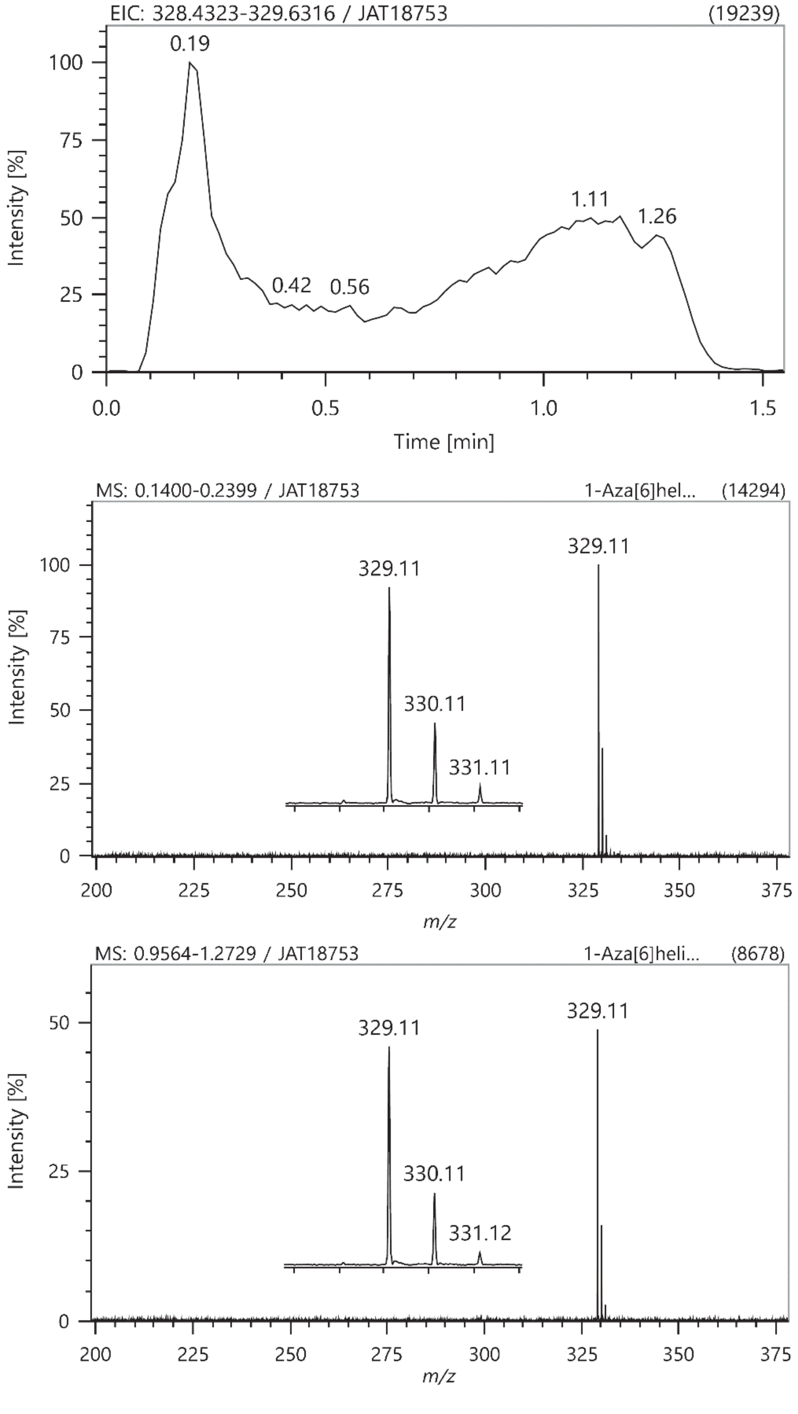

Fig. S10. Series of APFD spectra of 1-aza[6]helicene at various emitter potentials as indicated on the plots. The $[M+H]^+$ ion at *m/z* 330.1274 did appear at slightly lower potentials than the molecular ion at *m/z* 329.1196, the relative intensity of which also remained in the order of 2.5 %. Inserts show the molecular ion region with an expanded intensity scale to give a better estimate of the molecular ion peak intensity. Actual voltages applied to shield and cap are also noted on each spectral plot.

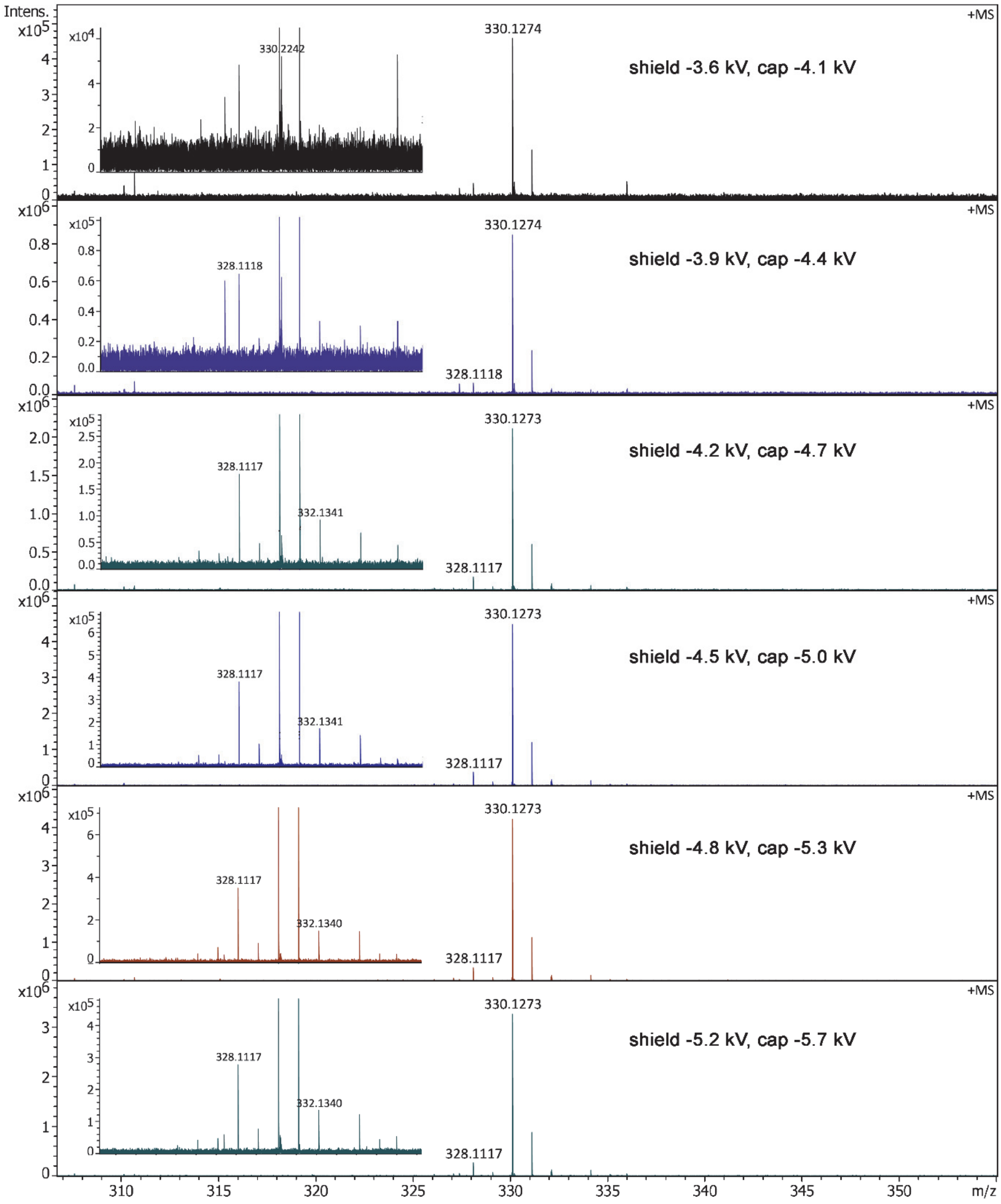

Supplement: Supplementary file 1 — Supplementary file1 (PDF 1841 KB) [file 216_2023_4652_MOESM1_ESM.pdf]
